# Acceptability of financial incentives and penalties for encouraging uptake of healthy behaviours: focus groups

**DOI:** 10.1186/s12889-015-1409-y

**Published:** 2015-01-31

**Authors:** Emma L Giles, Falko F Sniehotta, Elaine McColl, Jean Adams

**Affiliations:** Institute of Health and Society, Newcastle University, Baddiley-Clark Building, Newcastle upon Tyne, Tyne and Wear NE2 4AX UK; Newcastle Clinical Trials Unit, 4th Floor William Leech Building, The Medical School, Framlington Place, Newcastle upon Tyne, NE2 4HH UK; Centre for Diet & Activity Research, MRC Epidemiology Unit, University of Cambridge School of Clinical Medicine, Box 285 Institute of Metabolic Science, Cambridge Biomedical Campus, Cambridge, CB2 0QQ UK

**Keywords:** Qualitative focus groups, Acceptability, Financial incentives, Healthy behaviours

## Abstract

**Background:**

There is evidence that financial incentive interventions, which include both financial rewards and also penalties, are effective in encouraging healthy behaviours. However, concerns about the acceptability of such interventions remain. We report on focus groups with a cross-section of adults from North East England exploring their acceptance of financial incentive interventions for encouraging healthy behaviours amongst adults. Such information should help guide the design and development of acceptable, and effective, financial incentive interventions.

**Methods:**

Eight focus groups with a total of 74 adults were conducted between November 2013 and January 2014 in Newcastle upon Tyne, UK. Focus groups lasted approximately 60 minutes and explored factors that made financial incentives acceptable and unacceptable to participants, together with discussions on preferred formats for financial incentives. Verbatim transcripts were thematically coded and analysed in Nvivo 10.

**Results:**

Participants largely distrusted health promoting financial incentives, with a concern that individuals may abuse such schemes. There was, however, evidence that health promoting financial incentives may be more acceptable if they are fair to all recipients and members of the public; if they are closely monitored and evaluated; if they are shown to be effective and cost-effective; and if clear health education is provided alongside health promoting financial incentives. There was also a preference for positive rewards rather than negative penalties, and for shopping vouchers rather than cash incentives.

**Conclusions:**

This qualitative empirical research has highlighted clear suggestions on how to design health promoting financial incentives to maximise acceptability to the general public. It will also be important to determine the acceptability of health promoting financial incentives in a range of stakeholders, and in particular, those who fund such schemes, and policy-makers who are likely to be involved with the design, implementation and evaluation of health promoting financial incentive schemes.

**Electronic supplementary material:**

The online version of this article (doi:10.1186/s12889-015-1409-y) contains supplementary material, which is available to authorized users.

## Background

Poor engagement in health promoting behaviours is a key determinant of morbidity and mortality worldwide and results in substantial social, healthcare and economic costs [1]. Despite consistent efforts to encourage uptake of healthy behaviours, unhealthy behaviours remain common [2,3]. Developing effective methods to encourage uptake of healthy behaviours will result in substantial benefits to society as a whole.

Providing financial incentives to encourage healthy behaviours is one method of encouraging uptake of healthy behaviours. Health promoting financial incentives (HPFI) have been defined as cash or cash-like rewards or penalties, provided contingent on performance, or non-performance, of healthy behaviours [4]. This includes deposit contracts where individuals deposit their own money in advance and receive this back if they successfully change their behaviour, but forfeit the money if not [5]. Reviews in this area have found that HPFI can be effective in encouraging individuals to participate in health-promoting behaviours, although evidence is mixed in terms of effect size [6-16]. In particular, a recent systematic review of the effectiveness of HPFIs found that financial incentives were around 1.5 to 2.5 times more effective for promoting healthy behaviours than no intervention or usual care [4]. However, not only do financial incentives need to be effective and cost-effective, but they also need to be acceptable to potential recipients and wider society if they are to become a frequently used mechanism for achieving healthy behaviour change.

We recently conducted a systematic review bringing together both empirical and scholarly writing on the acceptability of HPFI [17]. The majority of the included papers were scholarly pieces rather than empirical evidence, and most of this scholarly writing appeared to lack an empirical evidence base. Where empirical evidence exists it is largely in the form of survey data rather than qualitative data providing detailed opinions on the acceptability of HPFI [18-27]. Thus, most of the debate within the literature on the acceptability of financial incentives appears to be unsubstantiated and represent the opinions of authors, rather than being underpinned by evidence. That said, the papers included in our systematic review argued that HPFI are acceptable under certain circumstances and that these are not substantially different from the circumstances under which most other public health interventions would be considered acceptable [17]. Health promoting financial incentives have been argued to be more acceptable when they are effective and cost effective [28,29]; when they provide an initial stimulus for behaviour change [30,31]; when they help to remove some of the financial barriers that individuals face when changing their health behaviours [32-34]; and when they foster behaviour change that can benefit wider society [35]. There is, nonetheless, a body of opinion that views HPFI as unacceptable under any circumstances. Reasons for this include concerns that some individuals may take-up an unhealthy behaviour in order to be eligible for HPFI, known as ‘gaming the system’ [36,37]; that HPFI can lower a person’s intrinsic motivation to change their behaviour [38,39]; that incentives can be coercive and force an individual to do something that they would not ordinarily do [32,40] and that HPFI schemes can be difficult to administer, implement and monitor [41-43]. That said, the debate on this topic is not necessarily evidence-based and is often contradictory [44].

Our review identified few previous qualitative studies on acceptability of HPFI. Those that have been conducted were restricted to alcohol, drug, and smoking cessation practitioners, managers and service providers, [21,20] pregnant women [26] and Maori, Pacific Islanders and low income groups [27]. Thus, growing, but still limited qualitative research on acceptability of HPFI has been conducted with a cross-section of participants, with limited research having been carried out in the United Kingdom (UK) [19,27,30,45-48].

We therefore conducted qualitative focus groups with members of the public to gain an in-depth insight into the factors related to acceptability of HPFI and preferred formats of HPFI.

## Methods

A qualitative methodology was adopted in order to provide in-depth empirical evidence on the acceptability of HPFI. Focus groups were chosen as they allow for a detailed discussion on the topic [49] and provided a forum for participants to reflect on their opinions towards HPFI based on the views of others [49]. Thematic analysis was undertaken on the data collected, with further details on the methodological approach provided below.

### Participants and recruitment

Focus group participants were recruited using a variety of methods. Members of two local databases of individuals, who had expressed an interest in taking part in research, were contacted (by database owners) with study information. As the contents of the databases were classed as confidential information by the list owners, we do not have information on how many people were approached or their characteristics. Eighty six individuals responded positively – mostly aged over 50 years, reflecting the make-up of the databases as a whole. Younger individuals were recruited through notices placed in newsletters, and on the staff websites of large, local employers. Again, we do not know how many individuals these invitations reached and so cannot calculate response rates. This resulted in 54 positive responses. The total available pool of participants was therefore 140, based on the number of responses to the recruitment notices. Potential participants were informed in advance that they would be assessed for eligibility and that this would determine whether or not they would be able to participate in the focus groups.

As is usually the case with qualitative research, we did not aim to recruit a representative sample; rather we aimed to conduct discussions with a cross-section of individuals. Aside from the age of the participants and their home postcode (to determine their social classification) we did not collect information on other socio-demographic, or health behaviour, characteristics of the sample.

A stratified sampling approach was used, [50] so that four of the eight focus groups contained ‘older’ participants (60 years or older) and four groups contained ‘younger’ participants (18–59 years). Within both the ‘younger’ and ‘older’ groups, further stratification based on affluence (social classification) was undertaken. Affluence of individuals was assessed using the ABC1C2DE classification of participant’s home postcodes. Post codes in ABC1 areas were classified as ‘affluent’, and post codes in C2DE areas were classified as ‘less affluent’ [51,52]. Thus two focus groups were ‘younger affluent’; two ‘younger less affluent’; two ‘older affluent’; and two ‘older less affluent’. Homogeneous focus groups were conducted to ensure that participants had shared connections which would help to promote an atmosphere of discussion and dialogue. This would also help participants to relate and connect to each other [53]. Having homogeneous groups helps to make the environment non-threatening so that participants are more likely to communicate their true attitudes and opinions [54,55].

### Data collection

We planned to conduct eight focus groups with around 8–12 participants per group. This number of individuals per group is large enough to generate discussion, but not too large that it impedes reticent group members from contributing [56]. We invited 12 eligible participants to each focus group hoping that at least eight would attend, selecting them on a first-come-first-served basis. Details of individuals who expressed an interest in taking part, but who were not invited to a focus group were kept on record in preparation for a separate phase of work, involving individual interviews. Only a minority of participants who were contacted declined to take part in the focus groups, usually because they were unable to attend on the chosen day or time. Focus groups continued until data saturation was reached – the point at which no new topics were being discussed.

Focus groups were held in an accessible University teaching room, located close to the centre of a city in North East England. This location was chosen to be convenient for the majority of participants.

Information sheets and consent forms were either emailed or posted to participants at least one week in advance of sessions, with email or telephone reminders made the day before. This was the only contact between the researcher and participants prior to data collection. The focus groups lasted approximately 60 minutes and were audio-recorded, with written consent being provided before each session commenced. At the end of each session, participants were provided with a verbal summary of the discussion and a written debriefing sheet, containing summary information of the research and the contact details of the researchers. As is routine, participants received £20 in high street shopping vouchers to remunerate them for their time, and were offered reimbursement of their travel expenses. This may have biased the sample towards those attracted by financial incentives. However, in practice, many of the participants had forgotten that they would receive a voucher for their time by the time that they attended the focus group. All data was held on password protected computers at the University. To preserve anonymity and confidentiality consent forms were stored separately from the audio recordings, and identifiers were assigned to the codes rather than naming individuals. Ethical approval was granted by Newcastle University Faculty of Medical Sciences Ethics Committee before data collection commenced.

A topic guide was developed for the sessions (see Additional file 1: appendix 1). This ensured that all topics of interest were covered during each focus group, but allowed for flexibility should new topics of relevance be raised by participants [57]. The researcher did not know why participants chose to take part in the research and did not ask in order to maintain objectivity throughout data collection and analysis. The four main discussion themes were: 1) whether participants had personally encountered financial rewards or penalties, 2) questions regarding the acceptability of incentives, 3) questions regarding the unacceptability of financial incentives, and 4) cross-cutting themes. Discussion was led by the same primary moderator (ELG) in all eight focus groups, and the moderator was careful not to influence discussions by giving their opinion. All researchers remained objective throughout data collection and analysis. A second moderator was present in all focus group sessions, who took written notes to ensure all key topics were covered.

### Data analysis

Focus group audio recordings were transcribed verbatim by an external company and checked by the primary moderator (ELG) for accuracy. Transcripts were then uploaded into NVivo 10 and subjected to thematic analysis [57-59]. The thematic analysis involved using the themes generated from our acceptability systematic review [17] as a starting point for coding, but with the additional option of coding words and paragraphs as new codes (nodes) should novel data emerge [50]. This approach was taken because we had some grounding in the likely themes that would arise in the focus groups, but we were unaware if additional themes would be discussed. The initial themes were: 1) the nature of fair exchange, 2) design and delivery, 3) effectiveness and cost-effectiveness, 4) recipients, and 5) impact on individuals and wider society [17]. Thus, the process of coding was a combination of inductive and deductive coding [60].

The first stage of the thematic coding was undertaken by one researcher (ELG) and involved close reading of the transcripts with words or phrases coded into one or more of the *a priori* themes, or coded as new nodes. Next, transcripts were re-read by ELG and all codes were checked for completeness. The third stage involved one researcher (ELG) reflecting on the thematic codes within each theme to ensure words and phrases had been assigned to the most relevant element. The final stage involved a ‘step back’ from the data, and the final themes were reflected upon in light of the research question, in order to determine a narrative. This final stage was undertaken by one researcher (ELG) and verified by a second (JA).Throughout data collection, analysis and write-up we considered the consolidated criteria for reporting qualitative studies (COREQ) checklist [60,61]. Transcripts were not returned to participants for correction. However, at the end of each focus group the researcher summarised the main themes that were discussed and gave participants an opportunity to correct the summary and change their opinions.

### Ethics

Ethical approval was granted by Newcastle University’s Faculty of Medical Sciences Ethics Committee, application number: 00670/2013.

## Results

Eight focus groups were conducted, involving a total of 74 individuals. Each group comprised between seven and 12 individuals. In total, 39 men and 35 women took part, 38 were ‘less affluent’ and 36 ‘affluent’ with 37 ‘older’ and 37 ‘younger’ participants. Overall, the majority of participants expressed a very negative opinion towards HPFI, compared to a minority who indicated that they thought HPFI were a useful catalyst for behaviour change. Those who would accept a HPFI personally, mostly said this was because they would particularly value the money received. Most participants had not personally received a HPFI and did not have knowledge of them in practice.

Largely, focus group discussions centred on the five original themes identified in our systematic review. There was some discussion of other topics which were coded as new themes. These are discussed below in turn, illustrated by verbatim quotations which clearly represent the theme.

### The nature of fair exchange

Fundamentally, most participants thought that HPFI were unfair. In particular, they thought it was unfair to reward ‘bad behaviour’ whilst not providing any such reward to those who follow healthy lifestyle recommendations of their own accord.

*“…it’s unfair to the healthy ones…”* [Focus Group 5, Younger, More Affluent]

*…”people being rewarded because you made a bad choice, and now you made a good choice, what’s my reward for making the right choice”* [Focus Group 7, Younger, Less Affluent]

Offering HPFI was considered particularly unfair to those who had found it difficult to change their behaviour without the use of incentives but had, nevertheless, managed to do so.

*“…you can always argue for all the people that have struggled with giving up smoking or struggled with weight loss and have managed to do that without the incentives… you could see it as a bit like well I’ve done it this way, why shouldn’t everybody”* [Focus Group 8, Younger, Less Affluent]

It was generally assumed that the costs of HPFI would be met by public ‘tax payers’ money, and it is likely that this would indeed be the case for any large-scale scheme in the UK. Some participants felt it would be unfair to use such money in a way that would only benefit a minority. Additionally, many participants regarded HPFI as a practice which was always considered unfair and amoral.

*“It’s unfair to everybody if it’s coming out of everybody’s pocket.”* [Focus Group 5, Younger, More Affluent]

*“Bribery is never going to be the answer … it does feel a bit like bribery”* [Focus Group 3, Older, Less Affluent]

### Design and delivery of incentive schemes

During discussions about the design and delivery of HPFI schemes, most participants discussed HPFI in terms that we have labelled as: ‘framing’ (e.g. as a reward for engaging in a healthy behaviour, compared to a reward for giving up an unhealthy behaviour); ‘administration and evaluation’ (who administers and evaluates the scheme and how), ‘funding sources’ (who funds the incentive scheme), and ‘design features’ (e.g. cash vs. shopping vouchers). This is in contrast to how we have previously conceptualised HPFI, in terms of the: direction of the incentive (reward or penalty); form (cash, shopping voucher or deposit); magnitude (incentive amount); certainty (whether the incentive is guaranteed or not); target (whether trying to change a behaviour is rewarded or just successful behaviour change); frequency (whether a reward is given for all behavioural instances or just some); immediacy (how soon the reward is provided); schedule (whether the same amount of incentive is provided each time); and recipient (who receives the incentive) [4]. This could partly be due to individuals being unable to ‘picture’ how HPFI schemes should be designed, due to the fact that few had personal experience of them. Some refused to answer questions on what HPFI schemes should look like because they fundamentally disagreed with the concept.

Where the framing of incentives was discussed participants said that it was preferable to reward people for making healthy choices and for showing long-term commitment to behaviour change. There was however some concern that HPFI would be stigmatising if participants received vouchers that were identifiable to the schemes, thus there was a preference for ‘anonymous’ rewards.

*“I think er you know it’s the whole notion of giving up something. It needs to be as if you’re making a different choice so you’re not going to spend money on cigarettes you’re going to spend it on the gym.”* [Focus Group 7, Younger, Less Affluent]

*“I agree that they need help but the help has to be done in a way that the incentive is not just about cash it’s also about them making a commitment to change their lives.”* [Focus Group 7, Younger, Less Affluent]

Where the incentive format was discussed most participants expressed a preference for reward schemes rather than penalties or deposit contracts. Often the rationale for preferring a reward format was related to accessibility issues. There was an opinion that deposit and penalty schemes would not be ‘open to all’, particularly if individuals had to find the money up-front in order to participate.

*“I think because of the current climate at the minute as well people wouldn’t be able to afford to put money up front.”* [Focus Group 4, Older, Less Affluent]

Largely, penalty schemes were disliked, for the same reason that deposit schemes were disliked: that they may not be available to all individuals, particularly those on lower incomes. Participants also wondered where deposited monies would go if they were not returned to individuals, preferring that money went to charity, rather than be divided between participants who had been successful at changing their behaviour. Schemes offering lottery tickets as rewards tended to be disliked by participants as they felt that individuals need a guaranteed reward should they be successful in changing their behaviour.

*“And I think a lot of the people you maybe want to help here won’t have the money to put up front.”* [Focus Group 3, Older, Less Affluent]

*“I do think that if there’s going to be a charge or some sort of penalty it’s gonna penalise people who are worse off, I’m not keen on that at all.”* [Focus Group 7, Younger, Less Affluent]

*“…secondly if somebody’s gonna be penalised where would that money be going?”* [Focus Group 6, Younger, More Affluent]

Similarly the preferred format of HPFI was vouchers rather than cash. Cash was argued to be open to abuse, such that recipients may spend cash rewards on the very behaviour that they were trying to change e.g. cigarettes. Whilst focus group participants reported that they would personally prefer to receive cash if they were to take part in a HPFI scheme, they were less trusting that other people would spend cash wisely and so preferred vouchers. A small number of individuals expressed a consistent preference for cash over vouchers because they felt cash could be used more flexibly than vouchers.

*“…like you say …* [with] *cash incentives though …* [it does make you] *think ‘oh well I know that’s, that’s what I should be doing with this money but I’ll just go and spend it on a packet of cigarettes or something.”* [Focus Group 6, Younger, More Affluent]

*“Probably cash myself… But vouchers for other people.”* [Focus Group 6, Younger, More Affluent]

In terms of the magnitude of incentives many participants were either unwilling or unable to provide a specific amount that they felt was acceptable for rewarding behaviour change. A rationale for providing ‘large’ incentives was that people would not find ‘small’ incentives sufficiently rewarding. Conversely, the rationale behind ‘small’ incentives was that it would help to make HPFI schemes more affordable, whilst also limiting abuse of the system. However, participants were unwilling to be more specific concerning what they meant by ‘large’ and ‘small’ incentives.

*“And money won’t, won’t make them change their lifestyle not, not unless you’re talking about large sums of money.”* [Focus Group 1, Older, More Affluent]

*“Yeah so however much erm I suppose money was dangled I suppose obviously there is a certain amount where it gets beyond and you think ‘oh right actually it really is worth it’.”* [Focus Group 6, Younger, More Affluent]

*“I think the higher the incentive as well the more abuse that’ll happen.”* [Focus Group 4, Older, Less Affluent]

Whilst it was agreed that individuals enrolled in the same behaviour change programme should all be provided with the same magnitude of incentive, it was suggested that HPFI could be tailored to individuals, such as asking individuals what type of reward they would prefer e.g. £50 in cash, £50 in vouchers, or a £50 gift. However, there was no discussion of how pragmatic or feasible such tailoring would be. Participants also said that HPFI schemes need to be convenient for people to enrol in and that they should correspond with the behaviour in terms of how long they are given to people, e.g. a short time for ‘simple’ behaviours, and a long time for ‘complex’ behaviours.

*“It depends how long the programme is and so on and events you know what behaviour you’re expecting people to change I mean ‘cause it’s related it’s got to be related to [it].”* [Focus Group 6, Younger, More Affluent]

*“Just personally I think if you’re gonna pay everyone that smokes just for example everyone who smokes a tenner [£10] not to smoke, I think you need to pay everybody that tenner [£10] a month ‘cause I think otherwise you’re gonna have people maybe wonder why they should bother if they only get a fiver [£5] and some others getting twenty quid [£20].”* [Focus Group 4, Older, Less Affluent]

*“It depends on what you have to do for it as well, that’s the thing if it’s gonna be a massive inconvenience so a big effort then you’re less inclined to do it I would think.”* [Focus Group 7, Younger, Less Affluent]

There was limited discussion on whether HPFI schemes should target outcome behaviours (e.g. stopping smoking) and/or process behaviours (e.g. attending smoking cessation clinics). Targeting process as well as outcome behaviours was argued to be preferable by some participants, to reward those individuals who genuinely tried to change their behaviours, but could not meet outcome targets.

*“You’ve got to be able to measure whatever you’re doing and sometimes people will make great efforts and yet don’t show any improvement.”* [Focus Group 5, Younger, More Affluent]

Lastly, two of the most discussed issues were the matter of administration and monitoring of HPFI schemes, and objectively verifying that recipients had changed their behaviour(s). Many participants said that HPFI schemes would need extensive and objective monitoring in order to confirm individuals were eligible to earn an HPFI.

*“Who’s going to monitor these things though…”* [Focus Group 6, Younger, More Affluent]

*“I think you need to be able to prove that you have achieved it.”* [Focus Group 7, Younger, Less Affluent]

*“I don’t think it would have to be up to the individual to report I think there’d have to be some sort of testing or weighing.”* [Focus Group 4, Older, Less Affluent]

### Effectiveness and cost effectiveness

One of the reasons why focus group participants found HPFI unacceptable may be partly related to them questioning the effectiveness of such schemes. Many participants questioned how effective HPFI schemes were, compared to discussing how effective and cost-effective they thought they were. Participants particularly wondered if HPFI would have a long-lasting effect on behaviour change, and whether behaviour would be maintained once incentives were withdrawn.

*“I would want to know if there is any research which has actually, good research which has actually proved evidence that any of these kind of these initiatives work.”* [Focus Group 3, Older, Less Affluent]

*“While you’re on your scheme you* [are] *possibly doing it. Whether it knocks on to actually a lifestyle change or a long term change I’m not convinced.”* [Focus Group 7, Younger, Less Affluent]

Some individuals were more pragmatic in their approach, and indicated that if HPFI could be shown to be effective and cost-effective over the long term, then it would be worth using them, even if they disagreed with them in principle.

*“I’ve been thinking about it and I would have said no, but I, I just think if, if nothing else works it’s worth giving it a try.”* [Focus Group 1, Older, More Affluent]

*“If it is efficacious, then of course the payment comes out in the balance doesn’t it?”* [Focus Group 5, Younger, More Affluent]

*“To see the efficacy of it, because I take a more pragmatic view than some of the other people here…That it all depends whether it works or not.”* [Focus Group 5, Younger, More Affluent]

There was also a lot of discussion concerning who would fund HPFI schemes. Many participants suggested that National Health Service (NHS) money could be used to fund HPFI, but only if over the long-term, money was saved through reduced expenditure on health care. This also led to discussion over whether the UK could afford to fund HPFI under the ‘current economic climate’.

*“The gentleman mentioned limited funding available, it’s probably not the best time to start giving people things for stuff like that.”* [Focus Group 5, Younger, More Affluent]

*“If they made a clear case that say they’d save money in the long run on certain things like if you managed to stop people smoking by giving them financial incentives and therefore the burden on the NHS was lessened.”* [Focus Group 6, Younger, More Affluent]

HPFI were said by some to be reactive rather than proactive, in that they do not address the underlying causes of unhealthy behaviours.

*“… incentives* [are like] *a bit of an Elastoplast in that it covers over but it doesn’t really get to the root of what causes it in the first place.”* [Focus Group 3, Older, Less Affluent]

### Recipients

Certain populations groups were identified by participants as more acceptable recipients of HPFI, particularly pregnant women and nursing mothers, and those on lower incomes.

*“I think there’s a big difference in giving breastfeeding mothers money to breastfeed ‘cause that’s got nothing to do with addiction.”* [Focus Group 3, Older, Less Affluent]

Participants did not think HPFI should be made available to those who they thought would ‘waste’ the money. Conversely, they felt it was important that HPFI did not inadvertently discriminate against certain social groups who may not be able to engage in the target behaviour.

*“But you’ve then got a runt of people who really no matter what you do for them, they’re always going to live badly and they’re always going to take what they can and, and give nothing back.”* [Focus Group 2, Older, More Affluent]

Additionally, some participants felt that HPFI were not appropriate for certain behaviours, such as encouraging healthy patterns of alcohol consumption.

*“But there’s something about paying someone to stop drinking that I think’s quite, I don’t know what the right word is it’s quite unsettling I think.”* [Focus Group 4, Older, Less Affluent]

### Impact on individuals and wider society

Some of the participants discussed an opinion that healthy behaviours are rewarding in themselves. For example quitting smoking results in better health and more disposable cash, even if a financial incentive is not provided. Participants were also concerned that if individuals were unsuccessful in changing their behaviours, and so did not receive an incentive, that they could become disillusioned and perhaps less likely to try to change their behaviour in future.

*“The fact that you’ve stopped smoking and you are not buying cigarettes should be a financial incentive to stop.”* [Focus Group 3, Older, Less Affluent]

*“You’re already healthy you’re leading such a marvellous life, that* [is] *its own reward in itself.”* [Focus Group 6, Younger, More Affluent]

*“It’s a funny one, because with the best will in the world people want to do it. And that just reinforces failure and if you know, if you can’t do it then you don’t get your incentive you know that’s a double failure isn’t it.”* [Focus Group 3, Older, Less Affluent]

However, HPFI were also felt by some participants to be a positive influence on individuals and wider society, when they were seen as an ‘added bonus’ and an initial catalyst for change, helping people to feel rewarded for their efforts; coming back to the need for HPFI schemes to be effective and cost-effective.

*“You know just to lose the weight but it’s a plus if you can get money.”* [Focus Group 6, Younger, More Affluent]

*“Yeah, as well as getting you going you actually feel ‘well I’m getting a pat on the back someone’s recognising my effort’.”* [Focus Group 6, Younger, More Affluent]

*“I think it can encourage people to change and if you just start that little bit that you know if they’ve had that incentive it might continue.”* [Focus Group 1, Older, More Affluent]

### ‘Other’ issues

Many focus group participants indicated that they would prefer education and peer support to HPFI. Participants felt these would be a more appropriate way to spend public money to encourage uptake of healthy behaviours. This was a particularly strong theme that was not identified in our previous review [17].

*“And it comes back to the point that people have made about education and I just think at that point it’s got to be somewhere where you think is it easier to put this money into changing people’s habits on a short term [basis] or is it better channelling that money into educating people and teaching people new types of habits?”* [Focus Group 6, Younger, More Affluent]

*“I think it would need to be accompanied by some sort of support with it.”* [Focus Group 4, Older, Less Affluent]

*“…a sense of belonging to a group or support, is quite important.”* [Focus Group 8, Younger, Less Affluent]

## Discussion

This is one of only a few qualitative studies on acceptability of HPFI [19,20,22,27,30,33,45-48,62-66] and one of only a limited number of empirical research studies on this topic including UK participants. In a cross-section of the public we found a range of views on the acceptability of HPFI which mostly reflected the themes we developed in a systematic review on this topic [17]. The results did not indicate that certain groups favoured (or not) incentives, e.g. older respondents having a more favourable attitude. These themes focused on: the nature of fair exchange, whereby incentives have to be fair to those who are already healthy, and fair in the sense of not discriminating against societal groups; that HPFI need to be shown to be effective and cost-effective before they are implemented wholesale; that certain individuals, such as pregnant women and low-income groups, are viewed as more acceptable recipients of HPFI; that HPFI can have positive impacts on individuals and wider society; and that HPFI should be designed and delivered in a way which is less open to abuse (e.g. vouchers rather than cash), and be tailored to individuals. The discussions also identified a range of possible methods for maximising the acceptability of HPFI in practice. These include providing reassurances that HPFI schemes will be monitored and evaluated; that they are effective and cost-effective; and that education and behaviour change support is provided alongside HPFI.

### Comparison to previous findings

Previous qualitative work on the acceptability of HPFI has reported similar findings in terms of monitoring and evaluation of HPFI schemes being important to recipients of HPFI. In interviews with pregnant women, Mantzari, Vogt and Marteau [26] found that vouchers were reported to help such women to stop smoking, but it was the monitoring of their behaviours in particular that facilitated and motivated women to quit smoking. Indeed, the robust monitoring of behaviours for those in the intervention group was said to have improved their chances of stopping smoking and thus attaining vouchers. This monitoring was not provided for the control group and so was stated as a reason for why fewer women in this group may have successfully stopped smoking, and therefore did not qualify for the vouchers. The monitoring was important in terms of effectiveness, but also in terms of it being a motivational experience and an important component of the intervention.

The issue of tailoring HPFI has been raised in previous qualitative findings [21]. Cameron and Ritter reported that alcohol and drug managers, practitioners and policy makers in Australia particularly advocated tailoring of incentives to better the particular circumstances of individuals [21]. Similarly our participants indicated that HPFI need to be tailored to individual preferences, such that one person may prefer £50 in cash, another, a £50 prize, and a third to place £50 in a deposit scheme. In addition, practitioners in Cameron and Ritter’s study expressed concerns about the use of monetary rewards, given that they could be open to abuse, and that HPFI were not an intervention approach that should be used on its own; rather HPFI should form part of a wider treatment plan. These issues were also discussed in our focus groups.

However, many of our focus group participants did not support the use of HPFI for behaviour change at all. Reasons, such as the potential for ‘gaming the system’, spending the cash on unhealthy behaviours (e.g. cigarettes) and HPFI being unfair to already healthy individuals, were particularly cited as contributing to the unacceptability of HPFI. This is in opposition to previous work, which suggests that HPFI were largely supported when offered for healthy eating behaviours, although ‘gaming the system’ issues have been mentioned in previous literature [29,67]. The difference here may be in part due to the very different population samples – a cross-sectional UK sample vs. a Maori and Pacific Islanders sample. It could also be that the focus groups with Maori and Pacific Islanders specifically focused on the use of HPFI for healthy eating, whereas our focus groups considered HPFI for healthy behaviours in general, and participants may not have been able to fully think through the pros and cons of HPFI for particular behaviours [68]. Additionally, support for HPFI was seen in focus groups with welfare service users in Australia, who indicated that HPFI for quitting smoking was acceptable to them, provided that individuals did not resort to deception to receive the money [23]. However, this positive endorsement was not reflected in the views from staff focus groups and interviews, where staff preferred non-cash HPFI rewards [23]. This reflects our findings that monitoring is considered particularly important.

Whilst there are contradictions to previous literature, our focus groups did identify findings that concur with previous research findings. In particular, our focus group participants argued that financial incentives could be discriminatory to certain individuals who are unable to change their behaviours in order to fulfil the criteria needed to obtain the outcome-based incentives [69]. Additionally, there was agreement that shopping vouchers are preferable over cash incentives; partly to overcome issues of ‘gaming the system’ and using the cash to fund unhealthy behaviours (e.g. buy cigarettes), which has been argued previously in the literature [20]. There was also a view that certain groups of individuals (and certain behaviours) were inappropriate targets for health promoting financial incentives, such as those with alcohol or drug issues. This viewpoint appears to be a common finding in relation to the acceptability of incentives [37,42,65]. Whilst the focus group participants were largely against financial incentives, there is evidence to suggest that as effectiveness of incentives increase, acceptability increases [47,62] and so this viewpoint may have been different had the focus group participants been provided with evidence of effectiveness.

Whilst there is limited qualitative evidence on the acceptability of HPFI in a UK context to-date, some of the findings that have been found in prior empirical studies are reflected in our findings.

### Strengths and limitations

We are confident that data saturation was reached, as few new topics emerged in the final few focus groups. Additionally, both the focus groups and the acceptability review found similar issues around the acceptability of HPFI, pertaining to: 1) the nature of fair exchange, 2) design and delivery, 3) effectiveness and cost-effectiveness, 4) recipients, and 5) impact on individuals and wider society. The focus groups found two additional themes which the acceptability review [17] did not, namely that HPFI schemes need to be monitored and evaluated, and that education is preferred over and above HPFI. Even though the acceptability review findings shaped the discussion guide for the focus groups, since new findings did emerge we are confident that the nature of the topic guide did not overly shape the discussions.

That said, given that the main themes arising from both the acceptability review and the focus groups were similar, we are confident that we have found empirical evidence which offers support for many of the arguments for and against HPFI purported in the literature. In particular, this empirical evidence supports many of the arguments we found in the scholarly writings in our acceptability review, although these original scholarly pieces tended to be based on opinion rather than evidence [36,42,70].

As is typical with qualitative research, we did not aim to recruit a ‘representative’ sample or generate generalizable results. However, the similarity of results from our systematic review and these focus groups gives credence to our findings. [17].

As a stratified approach to sampling was adopted, the views expressed on acceptability of HPFI are unlikely to be restricted to any particular population group. Indeed, we found that most participants – whether younger, older, affluent, or less affluent – did not view the use of HPFI as acceptable and that there were no strong differences in opinion between the groups. As we did not collect data on participant characteristics, aside from their age and home postcode, we are unable to detail the health behaviours of the participants (e.g. whether or not they smoked). Such characteristics may influence attitudes towards HPFI. For example, smokers who may be entitled to receive HPFI view incentives more favourably than non-smokers [19].

Focus group participants were not as forthcoming when asked to explain why they held certain opinions on HPFI, as they were when they were asked what those opinions were. This may because of the nature of group discussions, where participants may not feel comfortable explaining their views in front of others [71]. Additionally, participants were unsure of how effective HPFI schemes were and spent a great deal of time discussing this, possibly due to having limited knowledge and experience of such schemes. Their lack of knowledge on the effectiveness of HPFI may have contributed to their negative stance on HPFI for health promoting behaviours. In particular, participants often concluded that if HPFI schemes were ineffective then there is no reason to discuss acceptability. We did not provide information on effectiveness because we did not want to unduly influence the discussions. However, presenting evidence of effectiveness upfront, alongside providing examples where HPFI schemes have been used to good effect, may have helped reassure participants about their effectiveness and limit the focus they had on this issue. This may have provided more space for participants to fully explain the reasons behind their opinions and led to more favourable attitudes towards HPFI. A relationship between stated effectiveness and acceptability of HPFI has been demonstrated in quantitative research [47] and it would be interesting and useful to confirm and explore this relationship using qualitative methods.

### Interpretation of findings

The majority of participants held an unfavourable attitude towards HPFI. Mainly this negativity appeared to centre on the issue of trust, in that participants felt they could not trust other people to report behaviour change truthfully or use the money that they received as part of a HPFI scheme wisely. This seemed to influence which groups were considered ‘deserving’ or not of HPFI, e.g. vulnerable groups such as pregnant women were trusted to receive HPFI, but drug users were not. Ultimately, the majority of participants said that they could trust themselves not to abuse cash rewards, but that they could not trust other people. A variety of safeguards were therefore suggested to avoid abuse (e.g. objective monitoring and use of shopping vouchers instead of cash rewards). It could well be that careful consideration needs to be given to how the ‘target group’ for HPFI is defined. This may mean widening the definition of the ‘target group’ beyond those who might participate, to the public in general who all stand to benefit from improved population health. This may help to ensure ‘buy-in’ from across the population including those who are likely to fund any HPFI scheme through taxation.

Despite the majority view that HPFI were not acceptable, some participants were pragmatic, suggesting that if HPFI schemes were found to be effective and cost-effective in changing behaviours that they should be implemented. This concern for evidence of effectiveness and cost-effectiveness was common and suggests that implementation of a HPFI scheme should be accompanied by strong evidence of effectiveness and cost-effectiveness - as well as effective communication of this evidence to relevant stakeholders.

Participants also expressed a strong preference for alternative methods of behaviour change, particularly education and peer support. This reflects perhaps a need for greater feedback when individuals engage in behaviour change, with peer support providing verbal reassurance and positive motivation for individuals. The request for further education may mean that the way in which information is currently provided to individuals in behaviour change programmes (e.g. leaflets) is not recognised as education and may need to be re-designed in a way that ensures individuals recognise it as a form of educational help. Education and peer support may need to be built into any future HPFI scheme.

### Implications for policy, practice and research

Similar qualitative work is required in other stakeholder groups, in particular with policy-makers, given that these individuals will likely be involved in the design, implementation and evaluation of HPFI schemes in practice. How policy-makers view HPFI, how they would design HPFI schemes, and which recipients they would deem acceptable recipients, are key areas to explore in future research. Additionally, future research could explore the acceptability of financial incentives in members of the public for process as well as outcome behaviours (e.g. attending smoking cessation sessions vs. stopping smoking); and how individuals define ‘fairness’ in relation to HPFI. Lastly, future research could explore the potential for private sector companies to fund health promoting interventions in a UK context [72].

This qualitative empirical research has highlighted clear suggestions for how to design HPFI schemes, such that: positive rewards rather than negative penalties should be used; shopping vouchers should be provided rather than cash; HPFI have to be fairly implemented; HPFI schemes need to be closely monitored and evaluated; and education has to be clearly provided alongside HPFI.

## Conclusion

The evidence from qualitative focus groups with members of the public suggests that HPFI may be acceptable if schemes are closely monitored and evaluated; are proven to be effective and cost-effective; are delivered alongside education on how to change healthy behaviours and why; and are tailored to individuals. There was limited discussion on what HPFI schemes should look like, particularly because focus group participants wanted evidence of HPFI effectiveness and cost-effectiveness before they would discuss the acceptability of HPFI schemes. Overall, the majority of the focus group participants did not support the use of HPFI, although some were pragmatic, again saying that if HPFI are shown to be effective and cost-effective then they should be considered as a valid behaviour change intervention. If HPFI are to be used, more support was given for targeting vulnerable groups such as pregnant women and low-income groups. More research is needed into determining acceptability of HPFI in a wider range of stakeholders, particularly policymakers, who will be involved in designing and delivering HPFI schemes.

## Additional file

Additional file 1:
**A National Survey of Attitudes to Health-Promoting Financial Rewards.**
