# Supplementary material for: Acceptability of financial incentives and penalties for encouraging uptake of healthy behaviours: focus groups
Source: BMC Public Health. 2015 Jan 31;15:58. doi: 10.1186/s12889-015-1409-y (PMC4318173; doi:10.1186/s12889-015-1409-y)
Supplement: Additional file 1: — A National Survey of Attitudes to Health-Promoting Financial Rewards. [file 12889_2015_1409_MOESM1_ESM.pdf]

Supplementary Material

Appendix 1 – Focus Group Discussion Guide

A National Survey of Attitudes to Health-Promoting Financial Rewards

I. Introduction to the Research

- Welcome to the focus group
- This group will discuss the acceptability of financial rewards for health promoting behaviours, e.g. a cash reward in return for increasing physical activity.

II. Introduction to the Researchers

- I am Emma Giles and I am a researcher at the Institute for Health & Society at Newcastle University, with a background in marketing, public health and health behaviours.
- Introduce second moderator if applicable.

III. Ethical Issues

- Before we begin, has everyone brought their signed consent forms? (Collect forms)
- To remind you, this session will be audio recorded. If you object to this please say now.
- All data arising from this focus group will remain confidential and of use to the researchers. The data will be securely stored and any information you give will be anonymised. You are free to withdraw from this research at any time by contacting the research team.
- Can I also remind you that you are bound by confidentiality and for you not to repeat what is discussed outside of this room.
- A debriefing sheet will be provided to you at the end of the session providing you with the contact details of the researchers.

IV. Ground Rules

- Please contribute to the discussion. Everything you say is valued and there are no right or wrong opinions.
- If someone is talking please try not to talk over the top of them, as it is difficult to capture multiple voices on the audio recording.

- Please respect other people's opinions, even if they are different from your own.
- This discussion should take no longer than 90 minutes and could be much shorter.

V. Ice-Breaker'

- Can we introduce ourselves to the group. I'll start…

VI. Section One: Financial rewards

- Before we begin I would just like to provide you with a couple of examples of what we mean by financial rewards and losses.
- These incentives can be for many health behaviours: including smoking, healthy eating, vaccination and screening, physical activity, safe sun and sensible alcohol consumption.

- Can I just ask who has come across financial rewards or losses for smoking, dietary, vaccination, screening or safe sun behaviours? (In what context)

- Who has come across financial losses for these sorts of behaviour? (In what context)
- Who has come across lottery systems for these sorts of behaviours? (In what context)
- What do you think are the advantages of rewards, lotteries and penalties?
- What do you think are the disadvantages of rewards, lotteries and penalties?

VII. Section Two: What makes financial rewards acceptable?

- This second section will ask your opinions on whether you think financial incentives are acceptable and why. Please remember to think about rewards, penalties and lottery systems.

- Would you accept a financial incentive to change your behaviour?
- Why do you say that?
- Would you think it is acceptable to offer them to other people? Who? Why?
- Would financial rewards help you to change your behaviours? Can you explain?
- How fair do you think financial reward schemes are?

- Do you think financial reward schemes are fair to everyone? Can you provide examples of what you mean?
- How can financial reward schemes help individuals to achieve their behaviour goals?
- Should everyone be given financial rewards? Can you provide examples of what you mean?
- What do you think about financial rewards and long term behaviour change (e.g. for quitting smoking for two or more years)?
- What do you think is the effect of financial rewards on individual's motivation to change their behaviour?
- What advantages do financial rewards give to governments do you think?
- What advantages do financial rewards give to individuals do you think?

VIII. Section Three: What makes financial rewards unacceptable?

- This section will ask your opinions on whether you think financial rewards are unacceptable and why. Please remember to think about rewards, penalties and lottery systems.
- Do you think that individuals who receive financial rewards will be honest when they are reporting their behaviours to a researcher?
- For what reasons would you not accept a financial reward to change your behaviours?
- What do you think you would feel if you didn't manage to change your behaviour and so couldn't receive a financial reward?
- What do you think you would feel if you were penalised for not changing your behaviour?
- Do you think financial rewards discriminate against any particular (types of) individuals? Can you say who and why?
- What disadvantages do financial rewards give to governments do you think?
- What disadvantages do financial rewards give to individuals do you think?
- How cost-effective are financial rewards do you think?

IX. Section Three: Cross-cutting themes

- This section will ask your opinions on wider issues related to financial rewards.

- Where do you think financial rewards should be used? In which countries? Why?
- What are your feelings towards financial rewards?
- What are your feelings towards financial penalties?

- Do you think people should be penalised for unhealthy behaviours? Why?
- Do you think people should be rewarded for healthy behaviours? Why?
- Should everyone be entitled to the same incentives? Why? Why not?
- Do you think financial rewards can bring people together? Why? Why not?
- What format do you prefer for incentives and why?
- How much is an acceptable reward amount and why?
- How often would you need a reward and why?
- How would you prefer financial reward to be referred to, e.g. rewards, penalties, losses?

X. Questions/Comments?

- Thank you for participating in this focus group.
- We have discussed [quick summary].
- Does anyone have any comments or questions or wish to change their thoughts on incentives after hearing what others have said?
- Please take this debriefing sheet with you, and if you have any questions arising from your participation in this research please do contact me using the details provided.
- Thank you; here is the £20 voucher to thank you for your time. Please could you sign the sheet to say that you have received your voucher?
- If you have any travel expenses, please take one of these envelopes and complete the form as per the instructions inside.
- Thank you.
